# Spin-leap performance by cetaceans is influenced by moment of inertia

**DOI:** 10.1242/jeb.246433

**Published:** 2024-01-30

**Authors:** Frank E. Fish, Anthony J. Nicastro, Kaitlyn L. Cardenas, Paolo S. Segre, William T. Gough, Shirel R. Kahane-Rapport, Judy St. Leger, Jeremy A. Goldbogen

**Affiliations:** ^1^ Department of Biology, West Chester University, West Chester, PA 19383, USA; ^2^ Department of Physics and Engineering, West Chester University, West Chester, PA 19383, USA; ^3^ Hopkins Marine Station of Stanford University, Pacific Grove, CA 93950, USA; ^4^ Sea World, San Diego, CA 92109, USA

**Keywords:** Angular momentum, Breaching, Swimming, Spinning, Roll, Flippers

## Abstract

Cetaceans are capable of extraordinary locomotor behaviors in both water and air. Whales and dolphins can execute aerial leaps by swimming rapidly to the water surface to achieve an escape velocity. Previous research on spinner dolphins demonstrated the capability of leaping and completing multiple spins around their longitudinal axis with high angular velocities. This prior research suggested the slender body morphology of spinner dolphins together with the shapes and positions of their appendages allowed for rapid spins in the air. To test whether greater moments of inertia reduced spinning performance, videos and biologging data of cetaceans above and below the water surface were obtained. The principal factors affecting the number of aerial spins a cetacean can execute were moment of inertia and use of control surfaces for subsurface corkscrewing. For spinner dolphin, Pacific striped dolphin, bottlenose dolphin, minke whale and humpback whale, each with swim speeds of 6–7* *m s^−1^, our model predicted that the number of aerial spins executable was 7, 2, 2, 0.76 and 1, respectively, which was consistent with observations. These data implied that the rate of subsurface corkscrewing was limited to 14.0, 6.8, 6.2, 2.2 and 0.75 rad s^−1^ for spinner dolphins, striped dolphins, bottlenose dolphins, minke whales and humpback whales, respectively. In our study, the moment of inertia of the cetaceans spanned a 21,000-fold range. The greater moments of inertia for the last four species produced large torques on control surfaces that limited subsurface corkscrewing motion and aerial maneuvers compared with spinner dolphins.

## Teaser

**Summary:** The rate of spinning about the longitudinal axis of various cetaceans performing dramatic aerial leaps from the water varies with respect to their morphology, which affects the moment of inertia.

## INTRODUCTION

The proficiency of aquatic animals with respect to maneuverability is constrained by their morphology with regard to the flexibility of the body and the hydrodynamic characteristics and position of the control surfaces (e.g. fins, flippers, flukes, keels) influencing the animal's performance (Harris, 1936, 1937; Webb, 1984, 2004, 2006; Fish, 2002; Fish et al., 2003; Miklosovic et al., 2004; Weber et al., 2014; Fish and Lauder, 2017; Leahy et al., 2021; Segre et al., 2022). The morphology directly influences the control of the three rotational degrees of freedom of yaw, pitch and roll. Whereas turning using yaw and pitch has been the focus of much of the research on maneuverability in aquatic animals (e.g. Harris, 1936; Howland, 1974; Webb, 1983; Domenici and Blake, 1997; Fish and Nicastro, 2003; Weber et al., 2014; Segre et al., 2022; Downs et al., 2023), roll has received considerably less attention. This bias is due mainly to studies on organisms with gravity-centric orientations, hydrostatic stability, vertically oriented rudder-like median fins, and operation on a planar water surface. However, rolling around the longitudinal axis is important for turning by organisms that use laterally projecting control surfaces, have a center of gravity located near the center of buoyancy, and operate submerged in a three-dimensional environment (Fish, 2002; Goldbogen et al., 2013; Fish and Holzman, 2019; Segre et al., 2022). Particularly, marine mammals (cetaceans, pinnipeds, sirenians) will roll the body to turn by banking in order to facilitate use of their lateral control surfaces to hydrodynamically generate a centripetal force (Fish and Battle, 1995; Fish, 2002; Fish et al., 2003; Cheneval et al., 2007; Wiley et al., 2011; Segre et al., 2018; 2022). Underwater rolling is used by dolphins to increase the echolocatory insonification to the receiving areas to compensate for the asymmetrical and narrow echolocation beam (Wei et al., 2023). Rolling can also be used by the animal to change its energy state and linearly decelerate by transferring linear kinetic energy for translational speed to rotational kinetic energy as in the case of banked aerial turns (Giancoli, 1991; Lissaman, 2007).

**List of symbols and abbreviations**

*A*: area of control surface (m^2^)
*C*: drag coefficient
*D*: body diameter (m)
*I*: moment of inertia (kg m^2^)
*L*: body length (m)
*M*: body mass (kg)
*N*: number of complete spins
*R*: maximum radius of body (m)
*R*_D_: conformation constant for dorsal fin (kg m^2^ rad^−2^)
*R*_F_: conformation constant for caudal flukes (kg m^2^ rad^−2^)
*R*_P_: conformation constant for pectoral flippers (kg m^2^ rad^−2^)
*v*_s_: swimming speed (m s^−1^)
θ_R_: rotational performance coefficient (rad m^−1^, deg m^−1^)
τ: torque (N m)
ω_A_: angular speed of spinning underwater (deg s^−1^, rad s^−1^)
ω_SL_: maximum rate of aerial spin leaps (deg s^−1^, rad s^−1^)

Spinning can be considered a rolling maneuver in which rotations in the longitudinal axis of the body are produced. Such spinning behaviors are used for dismemberment of large food items (Helfman and Clark, 1986; Fish et al., 2007) and targeting prey underwater (Goldbogen et al., 2013). The most exaggerated of the spinning behaviors is displayed by the spinner shark, *Carcharhinus brevipinna*, and other carcharhinid sharks, especially the black-tipped shark, *Carcharhinus limbatus*, and by spinner dolphins, *Stenella longirostris*, and Clymene or short-snouted spinner dolphins, *Stenella clymene*. These species perform spectacular aerial leaps while spinning up to 7 times after clearing the water (Hester et al., 1963; Norris and Dohl, 1980; Perrin and Gilpatrick, 1994; Fish et al., 2006; Schwartz, 2013). The spinning behavior was inferred to be associated with the removal of remoras (Norris et al., 1994; Ritter, 2002; Ritter and Brunschweiler, 2003; Fish et al., 2006; Weihs et al., 2007; Schwartz, 2013).

The mechanics of the spinning leap by the spinner dolphins were modeled in Fish et al. (2006). The mathematical model demonstrated that angular momentum was generated while the dolphin was underwater. The resistive and driving torques generated by the dolphin's control surfaces were balanced to induce a low spin rate. Upon breaching the surface of the water, the torques became unbalanced with the driving torque dominating to produce an accelerated rate of spin. Analysis of the spinning leaps also indicated the body slenderness of the spinner dolphin with a fineness ratio of 6.3 enhanced spinning performance (Fish et al., 2006). A slender body would reduce the moment of inertia and foster greater angular acceleration than a thick body (Giancoli, 1991; Fish et al., 2006).

The present study was undertaken to investigate the relationship of the moment of inertia and the rate of spinning with respect to variation in size for cetaceans (i.e. dolphins and whales). We hypothesized that increasing body mass and girth in cetaceans would limit the number of aerial spins when leaping as a result of an increase in the moment of inertia. Cetaceans range in body length from 1.2 to 31.0 m (Nowak, 1999), but maintain a similar body design with a streamlined, fusiform shape. In the water, the body shape of cetaceans permits rolling maneuvers (Fish, 2002; Goldbogen et al., 2013; Segre et al., 2016). Many dolphins and whales leap or breach from the water into the air and are capable of varying degrees of aerial spinning (Au and Weihs, 1980; Whitehead, 1985; Au et al., 1988; Würsig et al., 1989; Weihs, 2002; Fish et al., 2006; Pearson, 2017; Halsey and Iosilevskii, 2020; Segre et al., 2020). A test of the hypothesis utilized data from video recordings of trained and wild dolphins, and biologging data from large whales executing aerial spins.

## MATERIALS AND METHODS

### Model for aerial spinning

The aerial capabilities of cetaceans are dynamically linked to their subsurface motions. The model of Fish et al. (2006), which was restricted to two species of spinner dolphins, assumes that animals establish a subsurface rotational equilibrium, balancing a propulsive rotational torque with drag torques acting on the control surfaces. As an animal emerges into air, those drag torques vanish as each control surface leaves the water, permitting the animal to angularly accelerate and thus increase its rate of rotation compared with its subsurface rate. For *Stenella longirostris*, a maximal 7 aerial spins in 1 s can result from a subsurface corkscrewing rate of 2 rev s^−1^. Corkscrewing is an underwater rolling maneuver in which the animal turns about its longitudinal axis (Fish et al., 2006). The model from Fish et al. (2006) also utilizes a rotational performance coefficient, defined to be θ_R_=ω_A_/*v*_s_, the ratio of the subsurface angular speed, ω_A_ (rad s^−1^) to the swim speed, *v*_s_ (m s^−1^). θ_R_ (rad m^−1^) represents a parameter that expresses the intuitive notion that the angular speed achievable while corkscrewing is directly proportional to the swim speed. θ_R_ is controlled by the animal and can vary from zero, when the animal swims in a straight line with no rotation about its longitudinal axis, to some maximum value permitted by the animal's morphology and regulated by the flexions of the control surfaces and, to a more limited extent, the torsion limit of the tailstock.

**Fig. 1.**
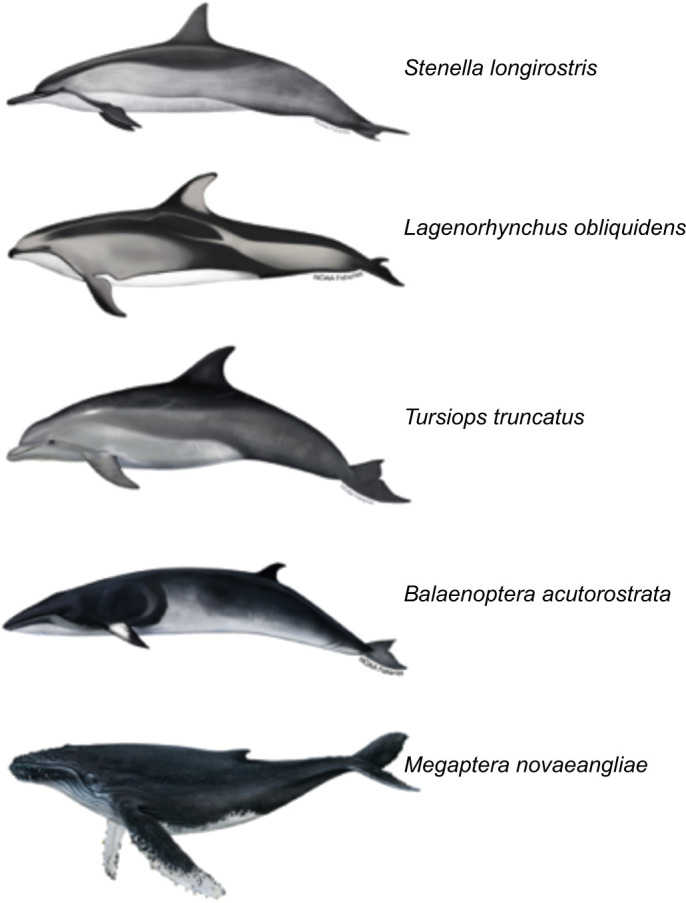
**Comparative body shapes of the cetaceans examined.** Images are arranged with respect to body mass from lowest to highest. Images are not drawn to scale.

**Fig. 2.**
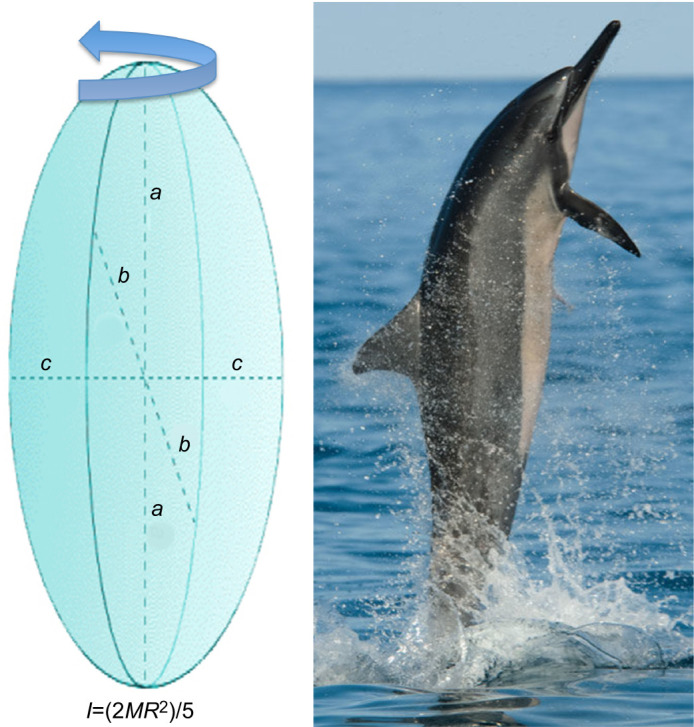
**Prolate spheroid and spinner dolphin (*Stenella longirostris*).** The dimension *a* was equivalent to the total body length. Dimensions *b* and *c* were equal and assumed to be equivalent to the maximum diameter of the cetacean. The equation for the moment of inertia (*I*) for the prolate spheroid is provided, where *M* is the body mass plus the added mass of the entrained water and *R* is the maximum radius of the body without the control surfaces.

In this study, we retained the fundamentals of the model developed in Fish et al. (2006) for spinner dolphins [*Stenella longirostris* (Gray 1828)], but (1) extend it to additional species of cetaceans, including other members of the Delphinidae with Pacific white-sided dolphin [*Lagenorhynchus obliquidens* (Gill 1865)] and bottlenose dolphin [*Tursiops truncatus* (Montagu 1821)], and members of the Balaenopteridae with the minke whale (*Balaenoptera acutorostrata* Lacépède 1804), and humpback whale (*Megaptera novaeangliae* Borowski 1781) (Fig. 1), (2) investigate the effect of control surface area on the number of executable aerial spins, (3) determine the rotational performance and spin index for each of the species studied, and (4) generalize the model to any animal with differing surface areas and numbers and locations of control surfaces.

The model developed by Fish et al. (2006) showed that for spinner dolphins with pectoral flippers, a dorsal fin and caudal flukes as control surfaces, the number, *N*, of complete aerial spins executed by an animal with total moment of inertia, *I*, swimming underwater at a speed, *v*_s_, and corkscrewing with an angular speed, ω_A_, is:
(1)

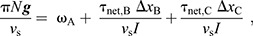
where ***g*** is gravitational acceleration (9.8* *m s^−2^), Δ*x*_B_ is the distance between the pectoral flippers and the dorsal fin and Δ*x*_C_ is the distance between the dorsal fin and the flukes. The moment of inertia (*I*) of a point-like body is the mass (*M*) times the square of the perpendicular distance to the rotation axis (Giancoli, 1991). For the bodies of the cetaceans, the moment of inertia was modeled as a three-dimensional prolate spheroid. The moment of inertia was calculated as *I*=(2*MR*^2^)/5 (Fig. 2), where *M* is the body mass plus the added mass in kg and *R* is the maximum radius of the body in m without the control surfaces. The added mass is the extra mass of fluid entrained to the body while in motion (Webb, 1975). For a whale-like body, the added mass is the body mass times the added mass coefficient (*k*_1_). According to Lamb (1932), for an ideal fluid, *k*_1_ is 0.059 and 0.045 for prolate ellipsoids with length to diameter ratios of 4.99 and 6.01, respectively. τ_net,B_ and τ_net,C_ are the net torques acting on the animal between the time the pectoral flippers begin to emerge from the water and the dorsal fin begins to emerge (stage B) and the net torque acting between the time the dorsal fin begins to emerge and the flukes begin to emerge (stage C), respectively (Fish et al., 2006). We can generalize Eqn 1 so that it permits us to adapt the model to cetaceans with nearly absent dorsal fin, such as with *M. novaeangliae*:
(2)

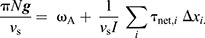


In general, Eqn 2 is useful in the analysis of the aerial maneuvers of animals with a more complicated array of control surfaces.

In our model, the resistive drag torques act on control surfaces as an animal initiates a subsurface corkscrewing maneuver. In this context, it is important to note that aerial spins are executable only if the animal's morphology permits large enough drag torques to be established. As drag torques are shed in a leap, a net torque results that produces an angular acceleration. The torques necessary to balance drag torques as the animal corkscrews, in turn, depend jointly upon the animal's morphology and physiology. Assuming that an animal's dorsal fin produces no torques other than a drag torque, corkscrewing at a constant angular speed ω_A_ involves balancing the hydrodynamic drive torque at the canted pectoral flippers and the drive torque produced at the flukes. This follows from the lack of a systematic torsion in the body while corkscrewing.

As shown in Fish et al. (2006), the resistive torques can be expressed as a function of ω_A_, the subsurface angular speed. For the pectoral flippers specifically, the torque produced by the pectoral fins is 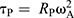, where *R*_P_ is a constant computed from the area of the pectoral flippers, their orientation relative to the longitudinal spin axis and other constants described in Fish et al. (2006). Similarly for the other control surfaces, the torque produced by the dorsal fin is 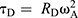, and for the flukes it is 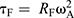. In addition to the resistive torque on the flukes produced by rotation about the animal's longitudinal axis, the oscillatory motion of the tail and flukes is responsible for producing the torque necessary to drive the animal forward. Because the lack of a systematic torsion in a corkscrewing cetacean implies that the total drive torque, τ_drive_, must be split equally between the hydrodynamic torques generated at the pectoral flippers and the drive torque produced by the flukes, we have the condition that:
(3)

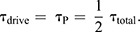
For an aerial spin to commence, τ_P_−½τ_total_≥0; that is:
(4)

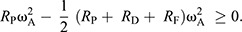
So:
(5)

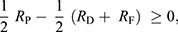
and finally:
(6)

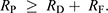
Eqn 6 represents the condition on a cetacean's morphology to be able to execute aerial spins.

As shown in Fish et al. (2006), the differential amount of resistive torque (dτ) produced by an element of area on a control surface, d*A*, a distance *R* away from the rotational axis is:
(7)

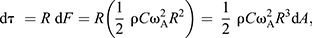
where the density of sea water, ρ, is 1025 kg m^−3^ and the drag coefficient, *C*, is 1.2 (Potter and Foss, 1975). For a constant corkscrewing rate ω_A_:
(8)

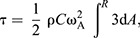
where the integration is over the area of the control surface. Thus, the condition for executing aerial spins in Eqn 6 can be rewritten as:
(9)

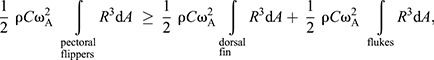
or
(10)

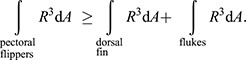
Thus, in the model's assumptions of a constant subsurface corkscrewing rate and rigid control surfaces, a cetacean's ability to execute aerial spins is determined solely by the geometrical form and positions of the control surfaces relative to the animal's longitudinal axis. As can be seen in either Eqn 6 or Eqn 9, the placement of the pectoral flippers sets the scale for the ability to spin: the torques produced by the pectoral flippers must exceed the sum of all other resistive torques. We note here that the number (*N*) of spins executable by a cetacean depends, as can be seen in Eqn 1, on the distribution of mass around the spin axis affecting moment of inertia besides the shape and placement of control surfaces.

### Spinning leaps of dolphins and whales

The spinning leaps of trained dolphins were performed at Sea World (SW; San Antonio, TX, USA; Movie 1) and the National Aquarium (NA; Baltimore, MD, USA; Movie 2). Two adult female *L. obliquidens* (SW) and two adult male and five female *T. truncatus* (NA) were used to study the behavior. Dolphins executed the spinning leaps in the center of large exhibit pools at each facility (Movies 1 and 2). Each pool was constructed with large underwater viewing windows. Two video cameras (Canon EOS 5D Mark III equipped with a Canon Zoom Lens EF 24–70* *mm, 1:2.8) were mounted on tripods and positioned to record the movements of the dolphins simultaneously below and above the water surface. The dolphins had been trained to perform the spinning leap on command. Leap height was measured as the vertical displacement of the center of gravity above the water surface. The center of gravity was assumed to be at a position of 0.4* L* (Fish, 2002). Morphometrics of the dolphins were supplied by the staff at each facility, including total body length (*L*), maximum body diameter (*D*), flipper width and span, fluke width and span in meters, and body mass in kilograms. The fineness ratio was calculated as the ratio of *L*/*D*. The research on the trained dolphins was approved by the West Chester University Institutional Animal Care and Use Committee (protocol no. 201201).

Data on the morphometrics and spinning leap performance for *S. longirostris* were obtained from Fish et al. (2006) for 858 animals that were collected as by-catch from the tuna purse-seine fishery in the eastern Pacific Ocean (S. Chivers, unpublished data). Morphometric data for one individual of *B. acutorostrata* and eight individuals of *M. novaeangliae* for *L* and *D* were determined from calibrated images using aerial drone photography according to the method of Gough et al. (2019). The maximum radius of the body (*R*) was one-half *D*. The mass (kg) of *B. acutorostrata* was computed according to Kahane-Rapport and Goldbogen (2018) with the equation log_10_*M*=3.091(log_10_*L*)+1.009. The mass (kg) of *M. novaeangliae* was calculated according to the equation *M*=1000(0.0158*L*^2.95^) from Lockyer (1976).

**Fig. 3.**
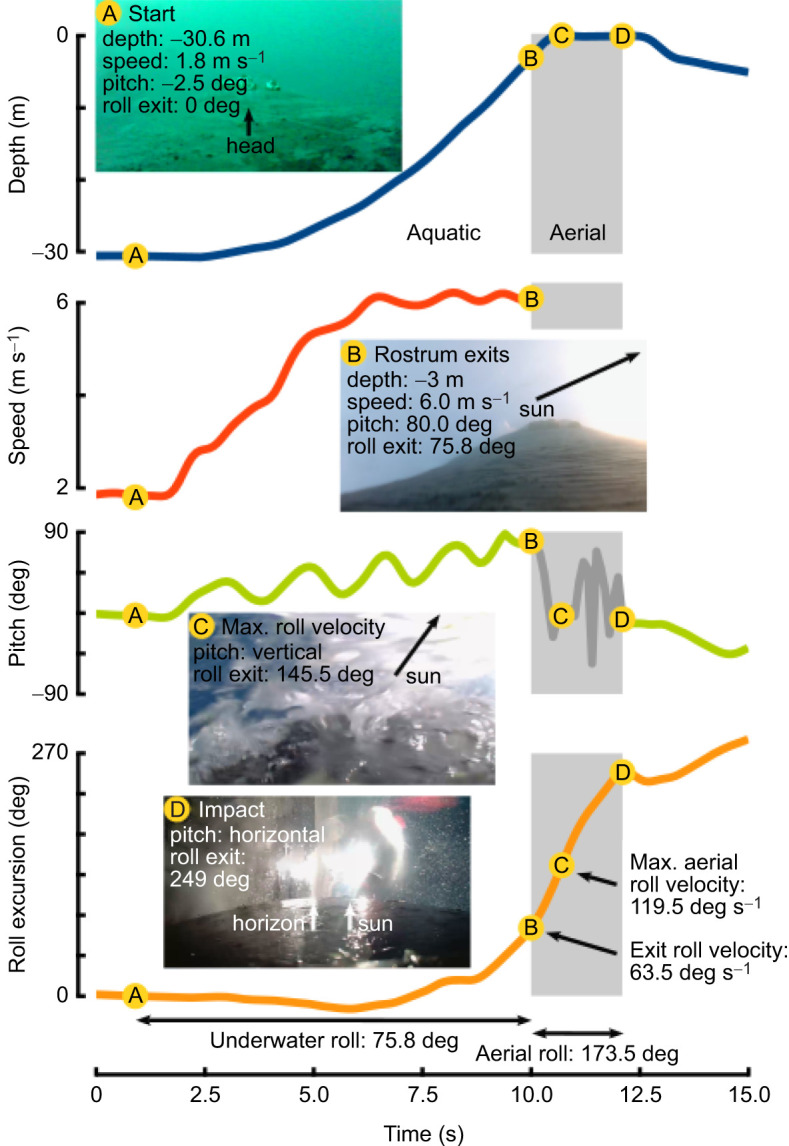
**Spinning performance of a humpback whale during a breach.** Biologging sensors measured depth (blue), speed (red) and pitch (green), and roll (orange) was measured using the gyroscope parallel to the axis of the long-axis of the body. Using the gyroscopes (as opposed to accelerometer-derived roll) allows for the measurement of roll when the whale is near vertical. Photos from the onboard cameras are shown as the whale begins its acceleration (A), as the rostrum emerges from the water (B), at the moment of the highest roll velocity (C) and as the whale hits the water and rolling stops (D). The arrows highlight different features of the whale and the environment, which act as a visual confirmation of the roll. Note, the speed measurement only works while the tag is underwater and therefore is not shown after the rostrum breaks the surface. Similarly, the accelerometer-derived pitch has a high level of error when the whale is out of the water (shaded) and should be interpreted with caution during this time. A video of this breach can be found in the supplementary material (Movie 3).

Spinning leap data for *B. acutorostrata* and *M. novaeangliae* were measured from suction cup-attached biologger recording tags (Gough et al., 2019; Segre et al., 2020). The tags were equipped with accelerometers, magnetometers, gyroscopes, depth sensors, hydrophones and on-board video camera (Fig. 3; see Segre et al., 2020, for specifications and deployment). The tags were placed on the dorsum of the animals and held by suction cups, which released for retrieval after a period of time. Breaches were only included for analysis where the suction cups did not slip or detach throughout the ascent, and where the orientation of the tag could be confidently estimated (Movie 3). The velocity of the whale in water was determined by the method described in Segre et al. (2020). The rotational velocities of the whales were measured with gyroscopes. Additional observations on spinning by *Megaptera* were obtained from YouTube videos (Table S1).

All research on the tagging and observations of baleen whales were conducted under approval of the National Marine Fisheries Service (permits 16111, 19116, 15271, 14809, 14682, 18059); National Marine Sanctuaries (MULTI-2017-007); Marine Mammal Protection Act (775-1875); Department of Environmental Affairs (RES2018/63); Nelson Mandela University animal ethics approval (A18-SCI-ICMR_001); Regional Directorate for Sea Affairs, Autonomous Region of the Azores (49/2010/DRA), and the Stanford IACUC.

## RESULTS

**Fig. 4.**
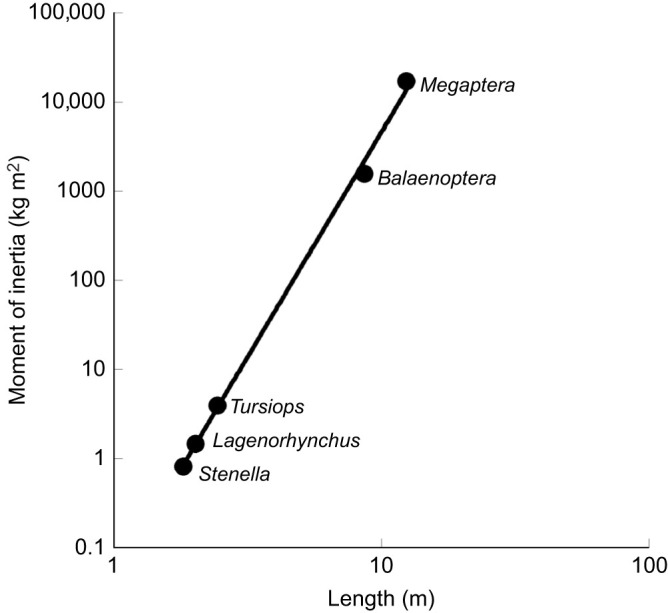
**Relationship between body length (*L*) and the moment of inertia (*I*).** This relationship was expressed by the equation *I=*0.041*L*^5.043^ with a correlation coefficient of *r*=0.997. Data are plotted on a logarithmic scale.

The average body dimensions and moment of inertia (*I*) are provided in Table 1. There was an overall size difference of about 6.8-fold between *S. longirostris* and *M. novaeangliae* in regard to both the body length and maximum radius. However, the difference in fineness ratio for the species examined was no greater than 25%. Body mass (*M*) showed a 443-fold difference for the species examined and *I* showed a 20,990-fold difference over the size range (Fig. 4).

**Table 1.**
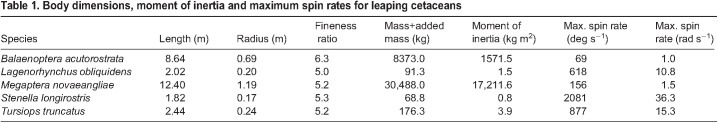
Body dimensions, moment of inertia and maximum spin rates for leaping cetaceans

**Fig. 5.**
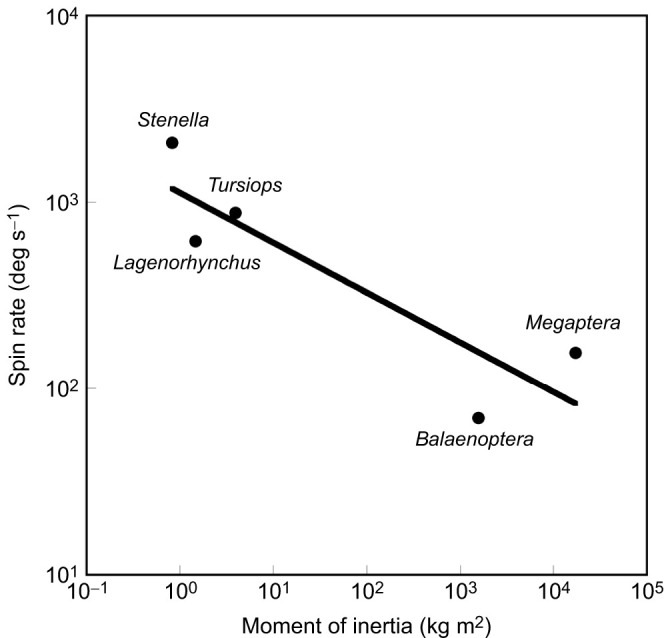
**The maximum rate of aerial spins (ω_SL_) with respect to the moment of inertia (*I*).** The relationship was expressed by the regression equation *I=*1123.6*L*^−0.268^ with a correlation coefficient of *r*=0.835. Data are plotted on a logarithmic scale.

**Fig. 6.**
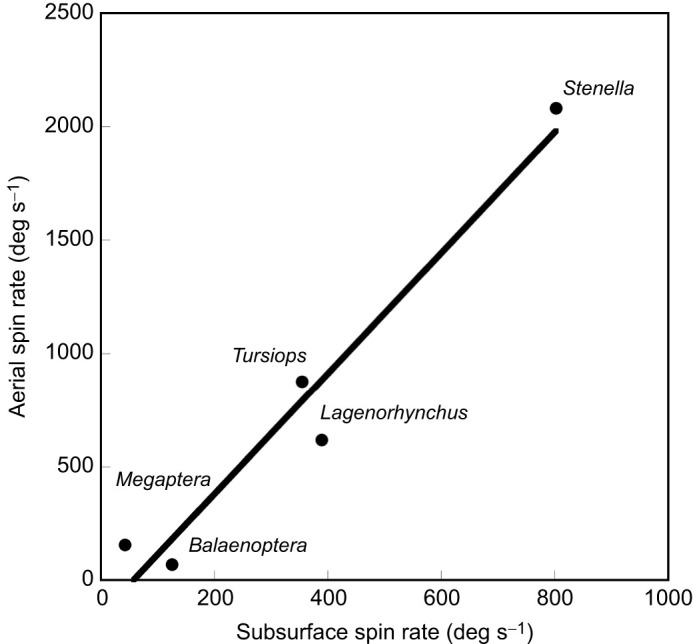
**Aerial spin ratio (ωSL) versus subsurface spin rate (ωA).** The linear relationship is expressed by the equation ωSL=2.66ωA−153.89 with a correlation coefficient of 0.973.

The number of aerial spin leaps examined was 5 for *B. acutorostrata*, 32 for *L. obliquidens*, 15 for *M. novaeangliae* and 38 for *T. truncatus*. The maximum rate of aerial spin leaps (ω_SL_) varied inversely with body size (Table 1; Fig. 5). The range of ω_SL_ varied from 156 deg s^−1^ to 2081 deg s^−1^, which represents a 30-fold decrease with increasing *M*. The highest maximum ω_SL_ was produced by *S. longirostris* with the lowest *I*, whereas the lowest ω_SL_ was produced by *B. acutorostrata* (Fig. 6). Despite the larger *M* and *I* of *M. novaeangliae* compared with *B. acutorostrata*, ω_SL_ for *M. novaeangliae* was 2.6 times greater.

**Fig. 7.**
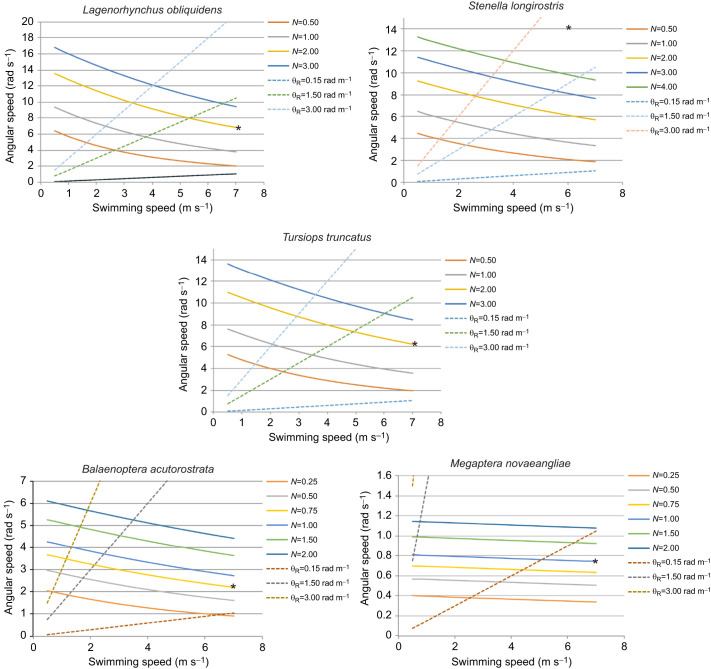
**The angular speed (ω_A_) while swimming underwater necessary to execute various numbers of complete spins (*N*; solid lines) over a range of swimming speeds (*v*_s_).** As a basis for comparison among the five species studied, the three dashed diagonal lines represent three values of the subsurface rotational performance coefficient, θ_R_ (in rad m^−1^), defined to be the ratio ω_A_/*v*_s_. The asterisks indicate the actual performance for each species studied; that is, the maximum number of spins observed corresponding to an animal's maximum swim speed. For example, for a *Lagenorhynchus obliquidens* individual, whose maximum swim speed is 7.3* *m s^−1^, a maximum of *N*=2 spins were observed (yellow solid curved line). The corresponding rotational performance coefficient is between 0.15 and 1.5 rad m^−1^, but can be computed more precisely using our model; those values appear in Table 2. For *L. obliquidens*, θ_R_=0.93 rad m^−1^, equivalent to 53 deg m^−1^ of subsurface travel. The solid curved lines in each graph were computed from Eqn 1 using morphometric data from measurements of individuals of each species. Once the dimensions of the body, the dimensions and location of the control surfaces, and the total mass have been measured, the individual's moment of inertia can be determined. Thus, for a particular swim speed, *v*_s_, the theoretical subsurface rotation rate, ω_A_, can be determined, which generates *N* spins. The observed maximum number of spins sets the rotational performance limit of each species.

The summarized results of the model for the hydrodynamics torques are shown in Fig. 7. The number of complete spins is dependent on the relationship between the swim speed and angular speed while underwater. High numbers of aerial spins by dolphins are achieved with higher angular and swimming speeds compared with low spin numbers as seen for the whales. With increasing swim speeds and lower *I*, more spins are possible for a given angular speed.

**Table 2.**
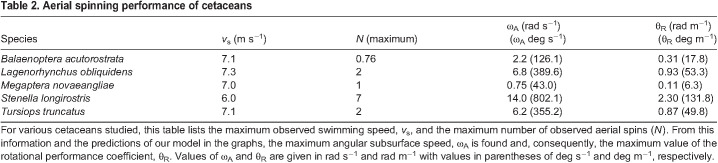
Aerial spinning performance of cetaceans

As indicated previously, the rotational performance coefficient, θ_R_, is controlled by the animal, but its maximum value is determined by the animal's morphology. The value of θ_R_ can be estimated via our model for spin-leap performance. That inference requires measurements of an animal's maximum swim speed and observations of the maximum number of spins executed by an animal. With these measurements, θ_R_ can be estimated.

To illustrate the application of the model in determining the rotational performance coefficient, θ_R_, consider the case of *T. truncatus*, whose maximum swim speed is 7.1* *m s^−1^ and which has been observed to execute up to 2 full aerial spins. The graph for *T. truncatus* in Fig. 7 shows the computed relationships between subsurface corkscrewing angular speed, ω_A_, and the animal's swim speed, *v*_s_, for various numbers of aerial spins. For *N*=2 spins and a maximum swim speed of 7.1* *m s^−1^, an asterisk (*) appears on the graph at that position. The corresponding value of ω_A_ is 6.2 rad s^−1^, according to the graph. Thus, θ_R_=ω_A_/*v*_s_=6.2 rad s^−1^/7.1* *m s^−1^=0.87 rad m^−1^, and that value is listed in Table 2. Note, though, that the values of ω_A_ listed in Table 2 were computed directly from the model using Eqn 1 and morphometric data, and not read from a graph.

The results for the five species studied appear in Table 2. Note that, apart from *S. longirostris*, the maximum swim speeds of the four other species in our study do not vary by more than 3% from the average of 7.1* *m s^−1^, yet *S. longirostris* individuals, swimming at a maximum swim speed of 6* *m s^−1^, about 15% slower than individuals of other species, achieve values of θ_R_ 2.5–21 times greater than the others. Of course, compared with *M. novaeangliae* and *B. acutorostrata* individuals, *S. longirostris* individuals have moments of inertia a few thousand times less, which certainly contributes to their subsurface performance. Still, *S. longirostris* individuals and those of *T. truncatus* and *L. obliquidens* possess moments of inertia that vary only by factors of 4.8 down to 1.8, respectively. Yet, both *T. truncatus* and *L. obliquidens* possess rotational performance ratios about 2.5 times smaller than those of *S. longirostris* individuals, implying that *S. longirostris* individuals can achieve greater flexions of their control surfaces compared with other dolphins.

Eqn 6 embodies the physical condition for any animal in our model to be able to execute aerial spins from subsurface corkscrewing: the resistive torques on the pectoral flippers must exceed the sum of the resistive torques on all the other control surfaces. Thus, in the case of *M. novaeangliae* and *B. acutorostrata*, smaller dorsal fins tend to enhance the ability to spin, but, as stated earlier, though the animal's ability to spin is enhanced, the number of spins executable is an interwoven function of the animal's moment of inertia, the size and placement of its control surfaces, its strength in powered motion, and its ability to regulate the flexion of its control surfaces. For *M. novaeangliae* and *B. acutorostrata*, their large moments of inertia are a primary limit to the number of executable aerial spins. From Table 1, we note that *M. novaeangliae*'s moment of inertia is 11 times that of *B. acutorostrata*, yet both whales are able to complete roughly one aerial spin (1 for *M. novaeangliae* and 0.76 for *B. acutorostrata*). Consistent with our model, *M. novaeangliae*'s performance is enhanced by its large, long flippers compared with those of *B. acutorostrata*, as illustrated in Fig. 1.

## DISCUSSION

Various animals cross the air–water interface. They do this to escape predators (Gudger, 1944; Fish, 1990; Connor and Heithaus, 1996), capture food (Würsig and Würsig, 1980; Martin et al., 2005; Reys et al., 2009; Johnston et al., 2018; Würsig and Whitehead, 2018), circumvent obstacles (Lauritzen et al., 2001; Kondratieff and Myrick, 2006), reduce the energetics of swimming (Hui, 1987; Au and Weihs, 1980; Weihs, 2002; Würsig and Whitehead, 2018), take a breath (Hui, 1989), communicate (Würsig and Würsig, 1980; Whitehead, 1985; Félix, 2004; Lusseau, 2006; Dunlap et al., 2008, 2010; Kavanagh et al., 2017; Dudzinski and Gregg, 2018; Werth and Lemon, 2020), play (Whitehead, 1985; Würsig and Whitehead, 2018), provide aerial vision (Würsig and Würsig, 1980; Würsig and Whitehead, 2018) and dislodge parasites (Hester et al., 1963; Ritter, 2002; Ritter and Brunschweiler, 2003; Fish et al., 2006; Weihs et al.,, 2007). Animals that plunge dive into the water use gravity to accelerate downward in an aerial phase but hydrodynamically decelerate with penetration into the high density and viscosity medium, which limits the depth attained (Sharker et al., 2019). Alternatively, animals leaping from the water use a high powered hydrodynamically dominated acceleration underwater to emerge into the air, where a gravity dominated aerial phase is associated with a deceleration that limits leap height (Chang et al., 2019; Halsey and Iosilevskii, 2020). Cetaceans (whales and dolphins) generally cross the air–water interface to become completely airborne when breaching and porpoising (Whitehead, 1985; Au et al., 1988; Hui, 1989; Fish and Hui, 1991; Pearson, 2017; Aguilar and García-Vernet, 2018; Halsey and Iosilevskii, 2020; Segre et al., 2020; Werth and Lemon, 2020; Xia et al., 2021; Milmann et al., 2023; Serres et al., 2023; Yu et al., 2023).

Superimposed on the ability to cross the interface and leap from the water is the ability to spin while breaching. The motion of a cetacean performing spinning leaps is a combination of translational and rotational motion. The center of mass of the animal moves along a ballistic trajectory that is dependent on the escape angle and escape velocity. The start of the animal's rotation around its longitudinal axis occurs underwater (Fish et al., 2006). Rotation is produced by an imbalance between driving torques and resistive torques from the control surfaces. Upon breaching through the water surface with only the flukes in the water, the hydrodynamic torque and resistive torque of the pectoral flippers disappear, as does the resistive torque of the dorsal fin. The drive torque from the flukes, which is greater than the resistive torque of the flukes, produces a torque imbalance (Fish et al., 2006). By conservation of angular momentum, the torque imbalance produces an angular acceleration, which increases the animal's rate of spin as it emerges from the water.

For bodies using paired control surfaces, spinning or rolling results from these appendages producing an imbalance between each of the two wings, fins or flippers. The imbalance is due to an asymmetrical pressure distribution and differential orientation of lift generation (Szurovy and Goulian, 1994; Segre et al., 2016; Fish and Lauder, 2017; Li et al., 2022). Such spinning maneuvers are used in aircraft acrobatics and military combat maneuvers as barrel rolls and slow rolls (Gunston and Spick, 1988; Szurovy and Goulian, 1994). For cetaceans, body mass is directly associated with moment of inertia, and the flipper area is responsible for the lift generated to affect a spinning moment (Segre et al., 2016). The spinning is initiated underwater, where the asymmetrical fluid forces are large enough to destabilize the body in the roll axis. Once airborne, conservation of angular momentum dominates and the spin rate increases (Fish et al., 2006).

The spinner dolphins and spinner sharks perform such aerial spinning maneuvers to dislodge remoras (Fish et al., 2006; Weihs et al., 2007; Schwartz, 2013). Spinner dolphins are able to perform leaps with up to 7 aerial spins (Fish et al., 2006) and an angular velocity of 4.6 Hz (F.E.F., personal observation). For both dolphins and sharks, the ability to execute multiple spins with high angular velocities in the air is dependent on body morphology. These dolphins and sharks have slender body profiles that provide a low moment of inertia.

Moment of inertia is the rotational analog of an inertial mass. Moment of inertia depends on how the mass is disturbed around the axis of rotation. With equivalent masses, a body with a large diameter will have greater rotational inertia and require a larger torque to start rotating than a more slender but longer body (Giancoli, 1991). The body shapes were similar with comparable fineness ratios of the other cetaceans examined, which would not ultimately affect the moment of inertia. Morphological differences affecting moment of inertia and thus spinning performance among the cetaceans were mainly mass, length and girth. Small, slender dolphins (*Stenella*, *Lagenorhynchus* and *Tursiops*) displayed greater rates of spin than the larger baleen whales (*Balaenoptera* and *Megaptera*) with greater moments of inertia.

Though Eqns 6 and 9 were developed and tested with data from studies of cetaceans, those equations, along with Eqn 2, can apply to any similarly shaped aquatic animal, living or extinct. As was discussed in the Introduction, spinning behavior can serve multiple functions (i.e. improved locomotion, maneuverability, play, dominance or aggressive display, alertness, acoustic communication, courtship display, dislodging ectoparasites), which are not restricted to cetaceans (Hester et al., 1963; Norris and Dohl, 1980; Norris et al., 1994; Fish et al., 2006; Weihs et al., 2007; Würsig and Whitehead, 2018). For example, remoras parasitize not only cetaceans but also sharks (Weihs et al., 2007; Schwartz, 2013). It is the expectation that the model can apply also to sharks known to execute aerial spins to rid themselves of attached remoras.

Differences in size affect performance for aquatic animals (Webb, 1975; Fish, 1998; Weber et al., 2014; Hirt et al., 2017; Gough et al., 2021; Segre et al., 2022). Although our study species have similar densities, the combination of smaller mass and diameter for the spinner dolphin, *S. longirostris*, gives a moment of inertia that is only 56%, 21%, 0.05% and 0.005% that of *L. obliquidens*, *T. truncatus*, *B. acutorostrata* and *M. novaeangliae*, respectively (Table 1). The increase in size reduced the maximum ω_SL_ and number of aerial spins that could be accomplished (Tables 1 and 2). The large whales (*B. acutorostrata* and *M. novaeangliae*) performed long-axis rolls prior to exiting the water. Whitehead (1985) noted that *M. novaeangliae* twists while leaving the water. We measured rolling velocity for the large whales with tags with gyroscopes but were unable to directly video record the whales when they were breaching and spinning. However, the videos from on-board tags suggest that, when employed, rolling can be initiated at different times. With shallow trajectories, the roll is often initiated immediately before the whale breaks the surface of the water: the extended flippers rotate contralaterally and the whale spins about its long axis. With deeper trajectories, the roll can be initiated much earlier. In both cases, the angular momentum continues the roll after the whale breaks the surface of the water (Fish et al., 2006).

Despite having a greater moment of inertia compared with *B. acutorostrata*, *M. novaeangliae* performed a maximum spin rate that was 2.3 times faster. The difference in spinning performance could be accounted for by the difference in flipper geometry. The flippers acting as control surfaces generate a lift force for the development of torque to initiate spinning underwater (Segre et al., 2016; Fish and Lauder, 2017). Members of the genus *Balaenoptera*, including the minke whale, use their flippers to roll at depth when targeting prey from below (Goldbogen et al., 2013; Segre et al., 2016). Maximum torque to spin is realized when one flipper generates a maximum upward lift and the other flipper generates a maximum downward lift (Segre et al., 2016). However, the dimensions of the flippers of *Balaenoptera* relative to the body size limits maneuvering performance (Weber et al., 2014). For a 14.4* *m long fin whale (*Balaenoptera physalus*), the planar flipper area and length are 0.1195 m^2^ and 1.48* *m, respectively (Segre et al., 2016).

Comparatively, a 9.02* *m long humpback whale (*M. novaeangliae*) has a planar flipper area and length of 1.02 m^2^ and 2.53* *m, respectively (Fish and Battle, 1995). Fish and Battle (1995) found that although *M. novaeangliae* was 37% shorter than *B. physalus*, the *M. novaeangliae* flipper had an area and length that was 88.3% and 41.5% larger, respectively, than for the *B. physalus* flipper. *Megaptera novaeangliae* uses its elongate flippers to perform aquabatic maneuvers. These maneuvers consist of tightly banked turns during bubble feeding and somersaults (Jurasz and Jurasz, 1979; Fish and Battle, 1995). Maneuvering by *M. novaeangliae* is also enhanced by the presence on the flipper of leading-edge tubercles that allow for increased lift production and delay of stall when operating at high angles of attack (Miklosovic et al., 2004; Fish et al., 2008; Fish, 2020). The geometry of the flipper of *M. novaeangliae* would compensate for the whale's large moment of inertia.

### Conclusion

The extraordinary leaps and aerial spins by cetaceans follow the fundamental laws of physics in regard to the moment of inertia and conservation of angular momentum. The rate at which each cetacean rotates about its longitudinal axis during aerial spinning leaps is dependent on the rate of spin underwater and the geometry of the body. The submerged spin rate is determined by the forces produced by the control surfaces that affect the whale's motion in roll prior to exiting the water. The large wing-like flippers of the humpback whale allow it to generate larger turning forces to perform greater spinning performance compared with a smaller whale. However in general, thin, small animals have higher rates of spin due to lower moments of inertia compared with large whales.

## Supplementary Material

10.1242/jexbio.246433_sup1 Supplementary information
