# Supplementary material for: Spin-leap performance by cetaceans is influenced by moment of inertia
Source: J Exp Biol. 2024 Jan 30;227(2):jeb246433. doi: 10.1242/jeb.246433 (PMC10914021; doi:10.1242/jeb.246433)
Supplement: Supplementary information [file jexbio-227-246433-s1.pdf]

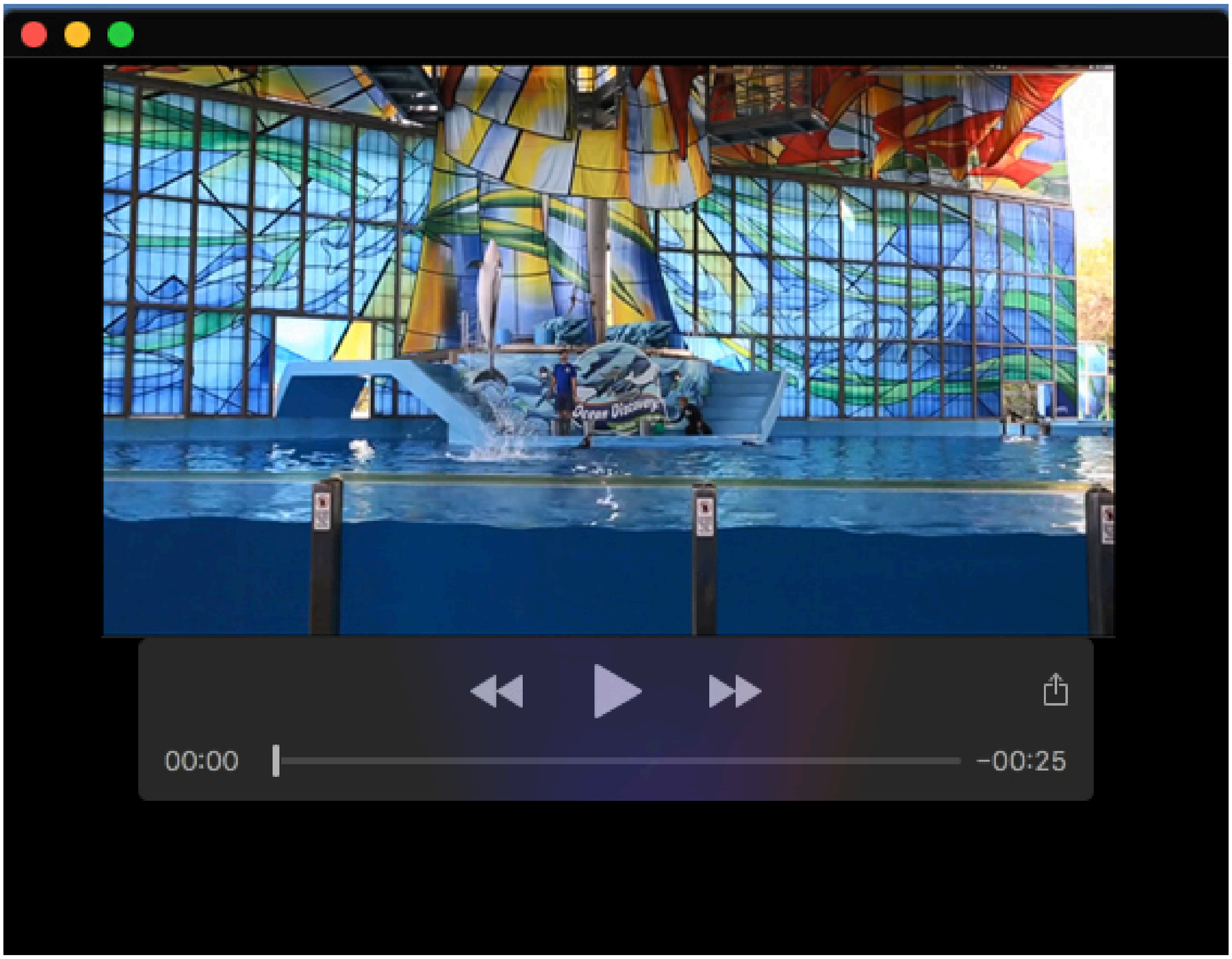

Movie 1. The video shows a Pacific white-sided dolphin (*Lagenorhynchus obliquidens*) during the underwater and aerial phases of a spinning leap. Underwater, the dolphin initiates a slow spin while swimming vertically toward the surface of the water. In the aerial phase, the dolphin leaps vertically about two body lengths above the water surface and increases its spin rate to 617.2 deg s^{-1} before falling back into the water.

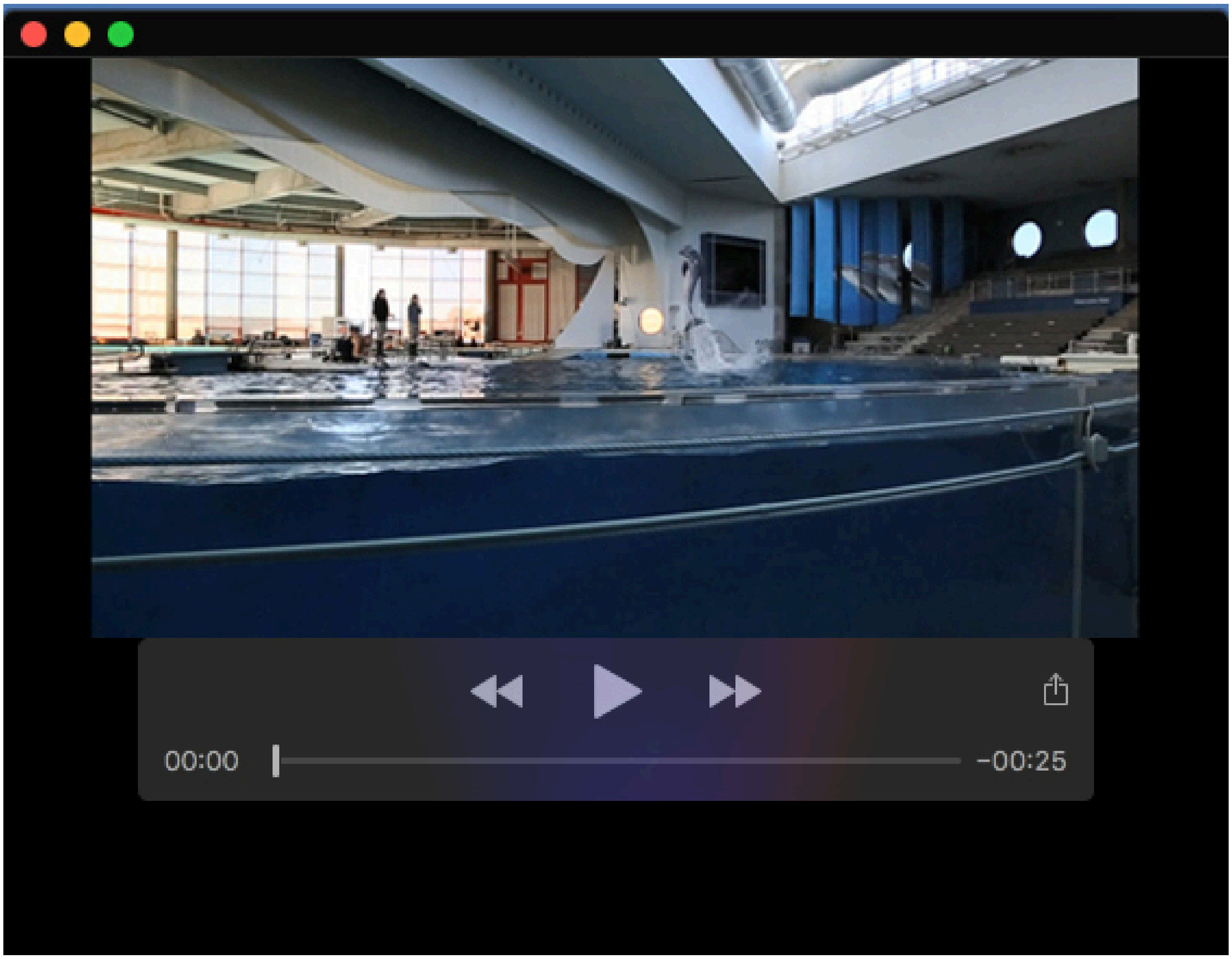

Movie 2. The video shows the spinning leap of a bottlenose dolphin (*Tursiops truncatus*). The dolphin rises to a maximum of height of 1.6 body lengths above the water surface. The dolphin contorts its body so that it flips over and re-enters the water head-first. The aerial spin rate was about 490.9 deg s^{-1}.

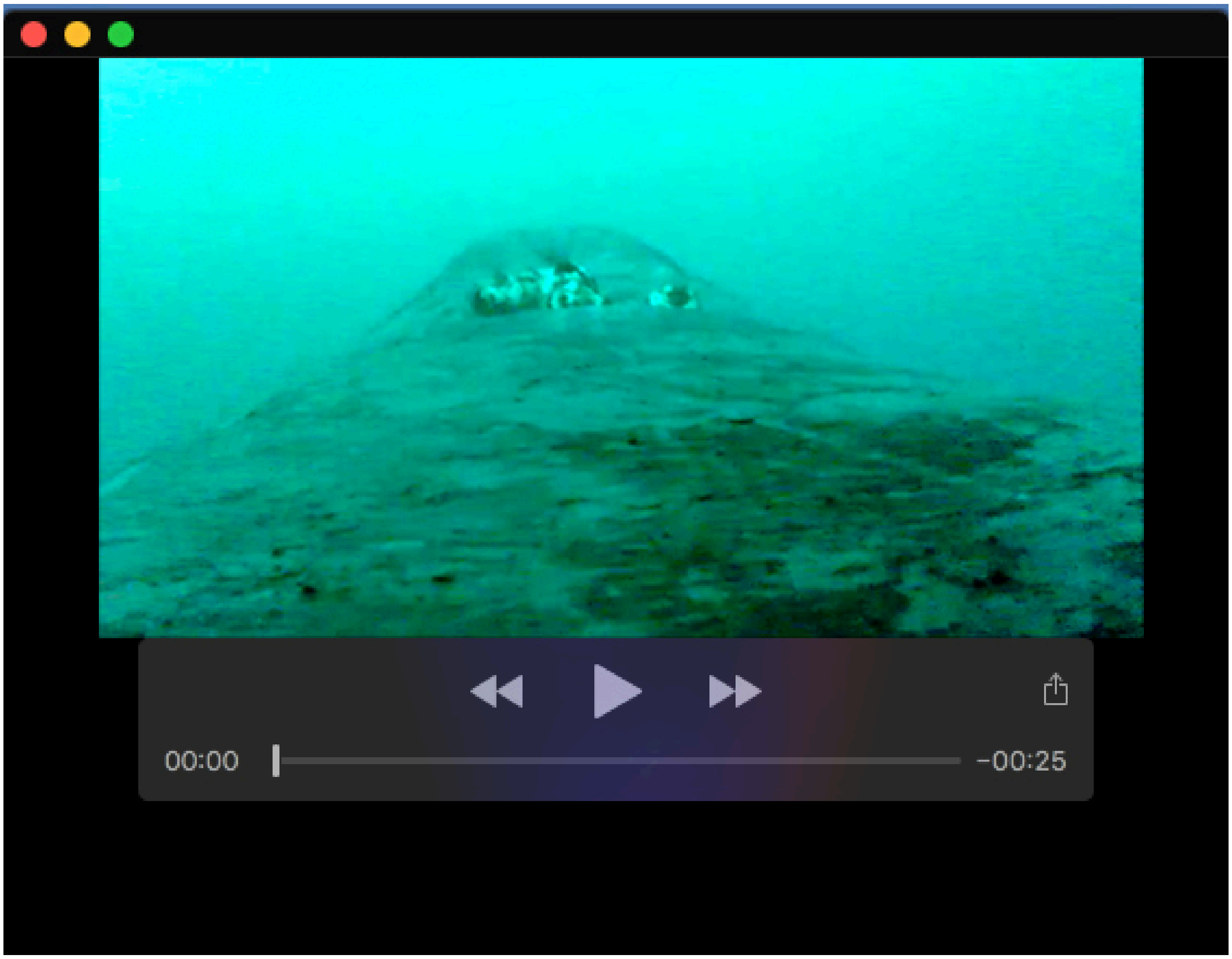

Movie 3. Video recorded from a suction cup tag attached to the dorsum of a humpback whale during a spinning breach.

Table S1. YouTube videos of humpback whale breaches

Title	url
40 Ton Humpback Whale Leaps Entirely Out of the Water! A Video by Craig Capehart	https://www.youtube.com/watch?v=fhflpUgxgm8
2013 Humpback Breach from Underwater Jones	https://www.youtube.com/watch?v=x6wZJ59nyqw
A Humpback Whale Breaches in Tofino, B.C. and Performs a Full 360-Degree Spin	https://www.youtube.com/watch?v=vpCv9-Sfnxl
Active Breaching Humpbacks: Mother "Big Momma" and her calf	https://www.youtube.com/watch?v=zdlrnMbx0o0
Incredible Humpback Whale Breach	https://www.youtube.com/watch?v=7NAKaSo19us
UNSEEN_ Whale breach near miss with swimmer	https://www.youtube.com/watch?v=A7ZON5ztSsc
Whale jumps out of nowhere during sight seeing tour	https://www.youtube.com/watch?v=zZTQngw8MZE
Whales breaching/ Huge jumps out of the water - Whale breaching	https://www.youtube.com/watch?v=hq-j9UQF30g
